# An Optimized Protocol for the Characterization of Zebrafish ApoB-Containing Lipoproteins Using the LipoGlo System

**DOI:** 10.21769/BioProtoc.5732

**Published:** 2026-07-05

**Authors:** Monica R. Hensley, Steven A. Farber

**Affiliations:** Department of Biology, Johns Hopkins University, Baltimore, MD, USA

**Keywords:** Lipoproteins, ApoB-containing lipoproteins, Zebrafish, LipoGlo, Cholesterol, Luciferase

## Abstract

Apolipoprotein B–containing lipoproteins (ApoB-LPs) transport lipids throughout the circulation and are closely associated with cardiovascular disease in humans. Many aspects of ApoB-LP biology remain elusive, often due to their indirect characterization through the measurement of plasma triglycerides and cholesterol. The conventional approach provides limited information on ApoB-LPs number and size distribution, essential features that influence cardiovascular disease risk. Additionally, drug studies have historically been limited to the use of mammalian research models, which are not suited for high-throughput experiments. Therefore, we generated a reporter system (LipoGlo) utilizing a luciferase enzyme (NanoLuc) fused to the C-terminus of the zebrafish (*Danio rerio*) ApoBb.1 protein. In metazoans, ranging from insects to humans, each ApoB-LP contains a single ApoB molecule, such that the luminescence emitted from these transgenic fish is proportional to the total number of ApoB-LPs. The LipoGlo zebrafish reporter generates a quantitative chemiluminescent signal that can be used in plate-based assays to measure lipoprotein quantities, a gel-based assay that can measure lipoprotein size distribution, and chemiluminescent microscopy that can, for the first time, visualize lipoprotein localization in a larval zebrafish. LipoGlo, combined with the amenability of zebrafish to genetic approaches, facilitates the rapid assessment of any gene or drug’s role in ApoB-LP molecular and cell biology. This protocol describes three optimized LipoGlo assays that facilitate ApoB-LP characterization with 100× less starting material than prior assays routinely used for mammalian lipoprotein analysis.

Key features

• Improves upon the methods from Thierer et al. [1].

• Lipoprotein profiles from individual zebrafish larvae that can simultaneously be genotyped for specific mutations.

## Graphical overview


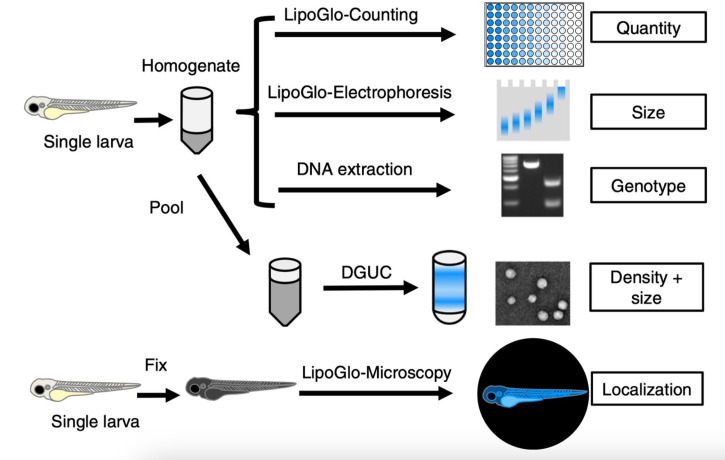


*LipoGlo zebrafish tissue homogenate can be used to measure the quantity, size, density, and localization of Apolipoprotein B–containing lipoproteins (ApoB-LPs). Reprinted/adapted from Thierer et al. [1]. © The Authors, some rights reserved; exclusive licensee, Springer Nature. Distributed under a Creative Commons Attribution Non-Commercial License 4.0 (CC BY-NC)* http://creativecommons.org/licenses/by-nc/4.0/

## Background

Apolipoprotein B–containing lipoproteins (ApoB-LPs) perform the vital role of transporting lipids throughout the circulation, being closely associated with cardiovascular disease, a leading cause of death worldwide [2]. Many fundamental aspects of ApoB-LP basic biology remain elusive [3–5]. Zebrafish and humans have many of the same key lipoprotein-related genes, including the major apolipoprotein genes *APOB, APOA1*, and *APOC2* [6,7]. They also share the conservation of the cholesterol ester transfer protein gene (*cetp*), which is a transporter of cholesterol esters and triglycerides between high-density lipoproteins (HDLs) and ApoB-LPs [8], a critical gene that is lacking in most rodents [9]. Thus, like humans, the zebrafish carry most of their cholesterol in their ApoB-LP fraction, whereas mice use HDL. The larval zebrafish are also ideally suited for high-throughput drug screens due to their small size (~5 mm in length), optically transparent development, and rapid organ development being completed by 5 days post fertilization (dpf) [10]. Despite the many advantages of using larval zebrafish, previous assays lack in sensitivity to profile ApoB-LP size and number in individual larvae [6,11,12].

In 2019, Thierer et al. described the LipoGlo reporter zebrafish system, in which genomic engineering was used to insert the coding sequence of a nanoluciferase (NanoLuc) reporter fused to the C-terminus of the zebrafish *ApoBb.1* gene [1]. The small (19.1 kDa) NanoLuc luciferase reporter generates a quantitative chemiluminescent signal through processing of its substrate, furimazine [13]. NanoLuc is ~100 times brighter than firefly luciferase and provides a robust signal-to-noise ratio that enables accurate detection at femtomolar concentrations [13]. Each lipoprotein contains one ApoB molecule, making the luminescence emitted proportional to the total number of ApoB-LPs [14].

In our original publication, we found that homozygous LipoGlo zebrafish emitted twice as much luminescence as heterozygous animals; however, in the years since, we found that this 2-fold increase is no longer consistently observed. We tested the hypothesis that the attenuated assay performance was due to premature depletion of LipoGlo substrate during the assay. In support of this hypothesis, we found that using 10× less tissue homogenate restores assay performance. ApoB-LPs come in a variety of sizes, with chylomicrons being the largest, and low-density lipoproteins (LDLs) being the smallest. The LipoGlo system can differentiate and quantify the levels of the different ApoB-LP size subclasses by subjecting tissue homogenates to a 3% native polyacrylamide gel electrophoresis (PAGE) followed by bioluminescent imaging. In our original publication, we used a commercially available DiI-labeled human LDL as a migration marker, but over time, we observed variations in the migration of the DiI-LDL, perhaps due to batch variations in LDL labeling. To better benchmark the LipoGlo Electrophoresis assay, we sought to develop a more consistent and validated migration marker. To achieve this, we used tissue homogenates from *ldlra^sd52^* mutants [15] that are enriched in small LDL particles and *apoC2^sd38^* mutants that have high levels of large very low density lipoprotein (VLDL) particles, confirmed by an orthogonal density gradient ultra-centrifugation (DGUC) assay to differentiate small and large particle fractions. Importantly, the DiI-LDL marker and the *ldlra^sd52^* ApoB-LPs consistently appear in the same DGUC fraction, indicating that, while the DiI label causes the resulting LDL to migrate at a slower rate on the PAGE gel, making it appear larger, it remains a useful standardization marker to address gel-to-gel variation [1]. In our original deployment of the LipoGlo electrophoresis assay using a PAGE gel to describe the sizes of ApoB-LPs, we included the signal from the loading well (zero mobility; ZM), thinking that it represented extremely large particles (e.g., chylomicrons). The protocol presented here suggests excluding the ZM signal because we now know from the DGUC analysis that it reflects aggregation of particles from a range of sizes that occurs when samples are loaded and run in the PAGE gel. In sum, we describe a series of LipoGlo protocol improvements to provide a more robust platform for studying ApoB-LPs in individual zebrafish larvae.

## Materials and reagents

**Biological materials**

Zebrafish stocks were maintained at 27 °C in a circulating aquarium facility. Adult zebrafish were fed once daily with ~2% body weight Gemma 300 (Skretting USA). Natural spawnings were used to obtain embryos, which were kept in embryo medium at 28 °C on a 14/10 h light/dark cycle until 7 dpf, unless noted otherwise. All experiments in this work were performed in larvae between 1 and 7 dpf. However, it should be noted that this protocol is optimized for 1–15 dpf [1] and has not been tested on earlier or later time points.

1. Zebrafish mutant line *ldlra^sd52^*, generated by the Yury Miller Lab, ZFIN ID ZDB-ALT-170913–3 [15]

2. Zebrafish mutant line *apoC2^sd38^*, generated by the Yury Miller Lab, ZFIN ID ZDB-ALT-151110–1 [6]

3. Zebrafish reporter line *Fus(ApoBb.1-nluc)*, generated in the Farber Lab, ZFIN ID ZDB-ALT-191218–4 [1]

**Reagents**

1. Ammonium persulfate (APS) (Bio-Rad, catalog number: 1610700)

2. Anhydrous calcium chloride (CaCl_2_) (Sigma, catalog number: C5670)

3. Boric acid (Sigma, catalog number: B0394)

4. Bromophenol blue (Sigma, catalog number: 114391)

5. Complete mini EDTA-free protease inhibitor cocktail (Sigma, catalog number: 11836170001)

6. Complete protease inhibitor cocktail (Sigma, catalog number: 11697498001)

7. Ethylene glycol-bis(2-amino-ethylether)-*N,N,N,N’*-tetraacetic acid (EGTA) (Sigma, catalog number: E3889)

8. Ethylenediaminetetraacetic acid (EDTA) (Sigma, catalog number: E9884)

9. Ethly 3-aminobenzonate methanesulfonate (tricaine, ms-222) (Sigma, catalog number: E10521)

10. HEPES (Goldbio, catalog number: H-400-100)

11. Low-density lipoprotein from human plasma, DiI complex (DiI-LDL) (Thermo Fischer, catalog number: L3482)

12. Low-melt agarose (Thermo Fischer, catalog number: BP165)

13. Magnesium sulfate anhydrous (MgSO_4_) (Sigma, catalog number: M7506)

14. Nano-Glo luciferase assay system (contains Nano-Glo^®^ luciferase assay substrate and Nano-Glo^®^ luciferase assay buffer) (Promega, catalog number: N1110)

15. OptiPrep density gradient medium (Sigma, catalog number: D1556)

16. Phosphate buffer saline (PBS) (Sigma, catalog number: 08057-12TAB-F)

17. Potassium chloride (KCl) (Sigma, catalog number: 7447-40-7)

18. Potassium phosphate monobasic (KH_2_PO_4_) (Sigma, catalog number: P8709)

19. Rain X original treatment (Walmart, catalog number: 800002242W)

20. Sodium bicarbonate (NaHCO_3_) (Sigma, catalog number: 1613655)

21. Sodium chloride (NaCl) (Sigma, catalog number: 1064040500)

22. Sodium hydroxide (NaOH) (Sigma, catalog number: 221465)

23. Sodium phosphate dibasic anhydrous (Na_2_HPO_4_) (Sigma, catalog number: S9763)

24. Sucrose (VWR, catalog number: BDH9308)

25. Tetramethylethylenediamine (TEMED) (Thermo Fisher, catalog number: 17919)

26. Tris Bas, (Thermo Fisher, catalog number: BP152-500)

27. Tris hydrochloride (Roche, catalog number: 10812846001)

28. Tween-20 (Sigma, catalog number: P1379)

29. 32% paraformaldehyde aqueous solution (PFA) (Electron Microscopy Sciences, catalog number: 15714)

30. 40% Acrylamide:Bis solution 19:1 (Bio-Rad, catalog number: 1610144)

**Solutions**

1. 20× embryo medium (see Recipes)

2. 1× embryo medium (see Recipes)

3. Tricaine (see Recipes)

4. 2× ApoB-LP stabilization buffer (see Recipes)

5. 2× ApoB-LP stabilization buffer (no sucrose) (see Recipes)

6. 0.5 M EGTA (see Recipes)

7. LipoGlo reaction buffer (see Recipes)

8. DNA lysis buffer (see Recipes)

9. 3% Native-PAGE gel (see Recipes)

10. 5× TBE (see Recipes)

11. 1× TBE (see Recipes)

12. Native-PAGE gel loading dye (see Recipes)

13. Native-PAGE gel imaging solution (see Recipes)

14. Low-density lipoprotein from human plasma (DiI-LDL) (see Recipes)

15. HEPES-buffered saline (HBS) (see Recipes)

16. 1 M HEPES buffer pH 7.4 (see Recipes)

17. 9% iodixanol solution (see Recipes)

18. 12% iodixanol solution (see Recipes)

19. 4% PFA (see Recipes)

**Recipes**

**1. 20× embryo medium**

**Table:** 

Reagent	Final concentration	Quantity or volume
NaCl	0.299 M	17.5 g
KCl	10.1 mM	0.75 g
CaCl_2_	19.6 mM	2.18 g
KH_2_PO_4_	3.01 mM	0.41 g
Na_2_HPO_4_	1.0 mM	0.142 g
MgSO_4_	19.8 mM	2.39 g
Milli-Q H_2_O	n/a	1 L
Total	n/a	1 L

Store at 4 °C.

**2. 1× embryo medium**

**Table:** 

Reagent	Final concentration	Quantity or volume
20× embryo medium	n/a	1 L
NaHCO_3_	0.83 mM	1.4 g
Milli-Q H_2_O	n/a	20 L

Store at room temperature.

**3. Tricaine**

**Table:** 

Reagent	Final concentration	Quantity or volume
Tricaine	19.5 mM	400 mg
1 M Tris	21 mM	2.1 mL
Milli-Q H_2_O	n/a	100 mL

Adjust pH to 7.0 with sodium bicarbonate and store at room temperature.

**4. 2**× **ApoB-LP stabilization buffer**

**Table:** 

Reagent	Final concentration	Quantity or volume
EGTA	40 mM	400 μL
Sucrose	0.58 M	1 g
Milli-Q H_2_O	n/a	Top up to 5 mL
Complete mini EDTA-free protease inhibitor	n/a	1 tablet

When making a 10 mL total volume, use one complete EDTA-free protease inhibitor tablet.

**5. 2**× **ApoB-LP stabilization buffer (no sucrose)**

**Table:** 

Reagent	Final concentration	Quantity or volume
EGTA	40 mM	400 μL
Milli-Q H_2_O	n/a	Top up to 5 mL
Complete mini EDTA-free protease inhibitor	n/a	1 tablet

When making a 10 mL total volume, use one complete EDTA-free protease inhibitor tablet.

**6. 0.5 M EGTA**

**Table:** 

Reagent	Final concentration	Quantity or volume
EGTA	0.5 M	9.3 g
Milli-Q H_2_O	n/a	50 mL
Total	n/a	50 mL

Store at room temperature.

**7. LipoGlo reaction buffer**

**Table:** 

Reagent	Final concentration	Quantity or volume
1× Phosphate Buffer Saline (PBS)	n/a	6.68 mL
Nano-Glo luciferase buffer*	n/a	1.1 mL
Nano-Glo luciferase substrate*	n/a	22 μL
Total	n/a	8 mL

*Reagents from Nano-Glo luciferase assay system.

Make fresh for each experiment.

**8. DNA lysis buffer**

**Table:** 

Reagent	Final concentration	Quantity or volume
NaOH	50 mM	2 g
Milli-Q H_2_O	n/a	500 mL
Total	n/a	500 mL

Store at room temperature.

**9. 3% Native-PAGE gel**

**Table:** 

Reagent	Final concentration	Quantity or volume
5× TBE	n/a	1.6 mL
Milli-Q H_2_O	n/a	5.7 mL
Acrylamide:Bis solution 19:1	40%	0.6 mL
Ammonium persulfate (APS)	10%	62.5 μL
TEMED	n/a	5 μL
Total	n/a	8.5 mL

**10. 5× TBE**

**Table:** 

Reagent	Final concentration	Quantity or volume
Tris base	446 mM	5.4 g
Boric acid	445 mM	2.75 g
0.5M EDTA pH 8.0	0.01 mM	2 mL
Milli-Q H_2_O	n/a	98 mL
Total	n/a	100 mL

Filter-sterilize and store at 4 °C.

**11. 1× TBE**

**Table:** 

Reagent	Final concentration	Quantity or volume
5× TBE	n/a	10 mL
Milli-Q H_2_O	n/a	40 mL
Total	n/a	50 mL

**12. Native-PAGE gel loading dye**

**Table:** 

Reagent	Final concentration	Quantity or volume
Sucrose	1.17 M	4 g
Bromophenol blue	3.7 mM	25 mg
1× TBE	n/a	10 mL
Total	n/a	10 mL

Store at -20 °C.

**13. Native-PAGE gel imaging solution**

**Table:** 

Reagent	Final concentration	Quantity or volume
5× TBE	n/a	1 mL
Nano-Glo luciferase substrate	n/a	2 μL
Total	n/a	1 mL

Make fresh for each experiment.

**14. DiI-LDL**

**Table:** 

Reagent	Final concentration	Quantity or volume
1× TBE	n/a	4 mL
Sucrose	0.1 g/mL	0.48 g
Human DiI-LDL	0.04 mg/mL	200 μL
Total	n/a	4.8 mL

Store aliquots at -80 °C.

**15. HBS**

**Table:** 

Reagent	Final concentration	Quantity or volume
NaCl	0.145 M	0.85 g
1 M HEPES buffer (pH 7.4)	0.1 M	10.0 mL
Milli-Q H_2_O	n/a	90 mL

**16. 1 M HEPES buffer (pH 7.4)**

**Table:** 

Reagent	Final concentration	Quantity or volume
HEPES	1 M	238.3 g
Milli-Q H_2_O	n/a	1 L

Adjust pH to 7.4 and store at room temperature.

**17. 9% iodixanol solution**

**Table:** 

Reagent	Final concentration	Quantity or volume
OptiPrep density gradient medium	n/a	1.5 mL
HBS	n/a	8.5 mL

**18. 12% iodixanol solution**

**Table:** 

Reagent	Final concentration	Quantity or volume
OptiPrep density gradient medium	n/a	2.0 mL
HBS	n/a	8.0 mL

**19. 4% PFA**

**Table:** 

Reagent	Final concentration	Quantity or volume
32% PFA aqueous solution	4.0%	12.5 mL
PBS	n/a	87.5 mL

**Laboratory supplies**

1. 96-well black OptiPlate (Perkin-Elmer, catalog number: 6005290)

2. Microseal B PCR plate sealing film (Bio-Rad, catalog number: MSB1001)

3. Template 96-well non-skirted PCR plate (USA Scientific, catalog number: 1402-9598)

4. 50 mL conical sterile polypropylene centrifuge tubes (Thermo Fisher, catalog number: 339653)

5. 15 mL conical sterile polypropylene centrifuge tubes (Thermo Fisher, catalog number: 339650)

6. 1.5 mL tubes (Thermo Fischer, catalog number: 3401-DLB)

7. 100 mm × 20 mm polystyrene Petri dishes (Sigma, catalog number: P5606-400EA)

8. Staples high-capacity heavyweight sheet protectors (Staples, catalog number: 15944)

9. Dumont No. 5 forceps (Sigma, catalog number: F6521-1EA)

## Equipment

1. Microplate-horn system (QSONICA, model: Q700MPXC)

2. Labnet Mini PCR plate spinner centrifuge (Spectra Services, model: C1000)

3. 4.9 mL OptiSeal^TM^ polypropylene tube, 13 mm × 51 mm (Beckman Coulter, catalog number: 362185)

4. 200 μL tapered round gel loading tip (USA Scientific, catalog number: 1252-0610)

5. Mini-PROTEAN^®^ Tetra cell casting module (Bio-Rad, catalog number: 1658021)

6. Mini-PROTEAN^®^ Tetra vertical electrophoresis cell (Bio-Rad, catalog number: 1658004)

7. Bausch & Lomb benchtop refractometer (Wazobia Scientific, catalog number: 33.467.10)

8. Beckman Coulter Optima XL 80K ultracentrifuge (Beckman Coulter, catalog number: A99833)

9. Rotor VTi65.2 (Beckman Coulter, catalog number: 362754)

10. Petri dishes 100 mm × 20 mm (Fisher Scientific, catalog number: FB0875711Z)

11. Zeiss AxioZoom microscope (Zeiss, model: V16)

12. Zeiss AxioCam MRm (Coastal Microscopes, catalog number: 51322K)

13. Zeiss BG40 IR blocking filter (Edmund Optics, catalog number: 16-367)

14. BioTek Syngery H1 microplate reader (Agilent, model: H1MF-SI)

15. Odyssey Fc imager (LI-COR Bioscience, model: Fc)

## Software and datasets

1. FIJI imaging processing software (NIH/http://fiji.sc/Fiji) [16]

2. Excel (Microsoft)

3. Prism (version 10.4.1 (532) GraphPad)

## Procedure

**A. Larval homogenate preparation for LipoGlo assays**

*Note: The homogenates produced in section A will be used in the LipoGlo counting assay (section B) and the LipoGlo electrophoresis assay (section C).*

1. Collect zebrafish embryos and keep n = 100 embryos in a 100 mm × 20 mm Petri dish with 70 mL of embryo media (EM) stored in an incubator at 28 °C with a light/dark cycle of 14/10 h.

2. Manually de-chorinate embryos still in their chorions with #5 forceps.

3. Pre-chill sonication horn with ice.

4. Pipette 50 μL of 2× ApoB-LP stabilization buffer into each well of an un-skirted 96-well PCR plate, keeping the plate on ice.

*Note: It is not essential to fill all of the wells of the plate; if using fewer wells, it is best to skip putting embryos into the outer wells of the plate, as these often have more issues sonicating fully.*

5. Add 1 mL of tricaine to the Petri dish to anesthetize the larvae before transferring them into the 96-well PCR plate.

*Note: It is important to follow the anesthetizing/euthanizing protocols approved for your lab by your institution’s Animal Care Committee. It is unknown whether fully euthanizing the larvae initially before plating and sonicating could lead to erroneous LipoGlo results. The goal is to minimize the time between death and sonication.*

6. To transfer the larvae, use a p200 pipette with the end of the pipette tip cut off and individually pipette one embryo in 50 μL of EM into each well, making the total volume in each well 100 μL. The pipette tip is cut so the embryos are not damaged during this process.

7. When the 96-well plate is full, use a Kimwipe to dry any droplets from embryo transfer before covering with a micro seal B plastic cover. Keep the plate on ice.

8. Add ice-cold water to the ice in the sonication horn chamber and measure to 17 mm in water depth. Remove all ice pieces before sonication.

9. Put the 96-well plate in the sonication horn, making sure there are no air bubbles trapped under the plate.

10. Use the following settings for sonication:

a. Amplitude 100%

b. Process time 30 s

c. Pulse-on 2 s

d. Pulse-off 1 s

11. After starting the protocol, pause sonication after every 4 pulses and rotate the plate 90° clockwise. One full round of the sonication protocol results in 16 total pulses: 4 pulses at each of 4 plate orientations.

12. After sonication, visually inspect the embryos/larvae and note wells that are not sonicated, as these will need to be eliminated from analyses. Wells that are not sonicated will still have a whole or mostly intact embryo/larvae and will be clearly visible compared to the sonicated samples.

13. Put the 96-well plate back on ice after sonication and use a micro-PCR plate centrifuge set to 2,500 rpm (500× *g*) for 15–20 s to spin down the homogenate. Return to ice following the spin.

14. If samples need to be genotyped:

a. In a new non-skirted 96-well plate, prepare the gDNA (genomics deoxyribonucleic acid) by adding 10 μL of 50 mM NaOH to each well, followed by 10 μL of the respective homogenate sample from the original 96-well plate.

b. Cover the gDNA plate with a micro seal B plastic cover and incubate at 95 °C for 20 min, cool to room temperature, add 1/10th volume of 1 M Tris buffer pH 8.0, and use 1–2 μL for PCR reactions.

c. For best PCR results, set up the PCR reaction the same day.

*Note: Due to shearing of the gDNA during sonication, PCR products 200 bp or smaller are recommended.*

15. For the most accurate LipoGlo Counting assay results, continue immediately to section B.

16. Alternatively, these homogenate plates can proceed to the LipoGlo electrophoresis assay in section C or be stored at -20 °C for future assays.

*Note: Do not exceed three freeze-thaw homogenate cycles, as it was shown in the original publication that three freeze-thaw cycles provide consistent luminescence [1]*.

**B. LipoGlo counting assay**

1. Pipette 76 μL of freshly prepared LipoGlo reaction buffer (see Recipe 7) into each required well of a flat-bottom 96-well black OptiPlate.

2. Add 4.0 μL of homogenate made in section A into each corresponding well of the black Optiplate.

3. Incubate on a horizontal shaker at low speed for 3–5 min at room temperature.

**Figure 1.**
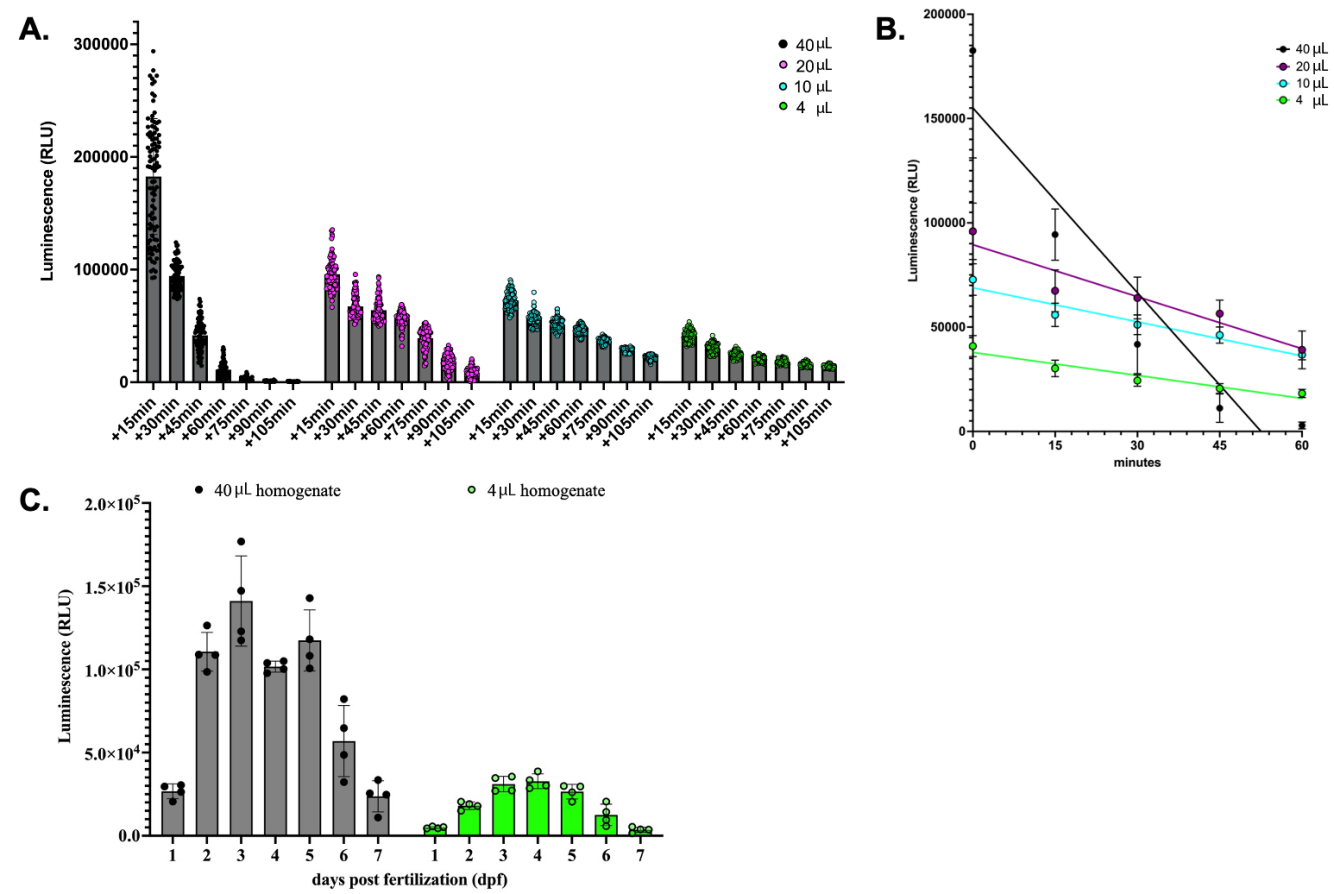
LipoGlo counting assay: optimizing tissue homogenate volume used in the assay. (A) LipoGlo counting data from assays with varying amounts of 4 days post-fertilization (dpf) larval homogenate (40.0, 20.0, 10.0, and 4.0 μL). Luminescence was measured in a plate reader (20 ms integration time) every 15 min for 1.5 h. N = 4, n = 384. (B) Linear regression of the luminescence signal vs. time for each volume of homogenate (40.0, 20.0, 10.0, and 4.0 μL) measured every 15 min for 1.5 h. Error bars indicate overall mean ± SD of three independent experiments. (C) LipoGlo counting assay using 40 or 4 μL of 4-dpf homogenate with luminescence measured over larval development from 1 to 7 dpf. N = 4 clutches, n = 384. Error bars indicate overall mean ± SD of three independent experiments.

*Note: Do not exceed 15 min of incubation time, as luminescence decreases over time (Figure 1A–C).*

4. Use a BioTek Syngery H1 plate reader with Gen5 software (version 3.05) and the following settings for plate reading:

a. Select all wells that have samples in them.

b. Select *Luminescence* and set the *Integration time* to 20 ms.

c. Select *Start* to read the plate.

5. Save data as an Excel file for subsequent data analysis.

6. Store the sealed 96-well plate of homogenate at -20 °C or on ice if continuing to the LipoGlo electrophoresis assay the same day.

*Note: Do not exceed three freeze-thaw homogenate cycles, as it was shown in the original publication that three freeze-thaw cycles provide consistent luminescence [1].*

**C. LipoGlo electrophoresis assay**

*Note: Before starting, pre-treat the mini-PROTEAN short plates front and back with Rain-X; this aids in the easy removal of the short plate for imaging purposes.*

1. Following Recipe 9 for Native-PAGE gel, mix the top three ingredients in a 50 mL conical tube.

2. Degas this solution for 30 min.

3. During the degassing step, assemble the mini-PROTEAN short plate and 1.0 mm mini-PROTEAN spacer plate in a Bio-Rad mini-gel casting frame and casting stand.

4. Add the required volume of 10% APS and TEMED to the conical tube and mix by gently inverting the tube; do not make bubbles (see Recipe 9).

5. Use a transfer pipette to fill each casting plate to the top and insert a multi-well comb with the desired number of wells.

6. Gels will set up at room temperature within 1 h and can be used immediately.

7. Equilibrate PAGE gel by running at 50 V for 30 min at 4 °C.

8. To prepare samples, start by thawing homogenate samples, prepared in section A, on ice.

9. Thaw an aliquot of DiI-LDL (see Recipe 14) on ice.

10. Pipette 12.0 μL of the desired homogenate samples into separate PCR tubes.

11. Mix the DiI-LDL aliquot by pipetting up and down, then pipette 12.0 μL of DiI-LDL into a separate PCR tube.

12. Add 3.0 μL of native PAGE loading dye (see Recipe 12) to all samples, including the DiI-LDL.

13. For loading PAGE gels, load 12.5 μL using a P20 pipette, but only pipette to the first stop of the pipette to avoid dispensing bubbles.

14. Set the electrophoresis power box to 50 V and run for 30 min.

15. After 30 min, increase the voltage to 125 V and run for 2 h.

16. Remove gels from the electrode assembly and take them to the imaging station.

17. Using a cassette opener, remove the short plate. The Rain-X treatment should help this process without damaging the gel on the large plate.

18. Pipette 1 mL of native-PAGE gel imaging solution (see Recipe 13) onto the gel and use a plastic sheet protector cut to the size of the large plate to spread the solution evenly over the gel.

19. Cover the gel, protecting it from light, and incubate at room temperature for 5 min before imaging.

20. Image using the Odyssey Fc (LICOR Biosciences) gel imaging system with Image Studio Software (version 6.2) and image in the chemiluminescence channel for 2 min (NanoLuc detection) and then the 600 channel for 30 s (DiI-LDL standard detection).

21. Export the raw images in a .zip file for subsequent analysis.

**D. Density gradient ultra-centrifugation (DGUC) assay and analysis**

1. Collect zebrafish embryos and keep n = 100 embryos in a 100 mm × 20 mm Petri dish with 70 mL of embryo media (EM) stored in an incubator at 28 °C with a light/dark cycle of 14/10 h.

2. Embryos still in their chorions need to have the chorions removed manually with #5 forceps.

3. Pre-chill sonication horn with ice.

4. Pipette 50 μL of 2× ApoB-LP stabilization buffer into each well of an un-skirted 96-well PCR plate, keeping the plate on ice.

*Note: It is not essential to fill all wells of the plate; if using fewer wells, it is best to skip putting embryos into the outer wells of the plate, as these often have more issues sonicating fully.*

5. Add 1 mL of tricaine to the Petri dish to anesthetize the larvae before transferring them into the 96-well PCR plate.

6. Use a p200 pipette with the end of the pipette tip cut off and individually pipette one embryo in 50 μL of EM into each well, making the total volume in each well 100 μL. The pipette tip is cut so the embryos are not damaged during this process.

7. When the 96-well plate is full, use a Kimwipe to dry any droplets from embryo transfer before covering with a micro seal B plastic cover. Keep the plate on ice.

8. Add ice-cold water to the ice in the sonication horn chamber and measure to 17 mm in water depth. Remove all ice pieces before sonication.

9. Put the 96-well plate in the sonication horn, making sure that there are no air bubbles trapped under the plate.

10. Use the following settings for sonication:

a. Amplitude 100%

b. Process time 30 s

c. Pulse-on 2 s

d. Pulse-off 1 s

11. After starting the protocol, pause sonication after every 4 pulses and rotate the plate 90° clockwise. One full round of the sonication protocol results in 16 total pulses: 4 pulses at each of 4 plate orientations.

12. After complete sonication, visually inspect the embryos/larvae and note wells that are not sonicated, as these will need to be eliminated from analyses.

13. Put the 96-well plate back on ice after sonication and use a micro-PCR plate centrifuge set to 2,500 rpm (500× *g*) and spin for 15–20 s to spin down the homogenate. Return to ice following the spin.

14. Pool together n = 15 homogenate samples into one 1.5 mL microcentrifuge tube.

15. Spin down the microcentrifuge tube at 6,000× *g* for 5 min.

16. During the 5-min spin, prepare a new microcentrifuge tube containing 500 μL of OptiPrep density gradient medium.

17. Following the 5-min spin, pipette 1 mL of the resulting supernatant into the tube prepared in step D16, yielding a 20% iodixanol solution.

18. In a 4.9 mL OptiSeal tube, add 1.5 mL of 9% iodixanol (see Recipe 17) in HBS solution (see Recipe 15).

19. Underlay with 1.5 mL of the 12% iodixanol solution (see Recipe 18) using a p1000 tip fitted with a tapered gel loading tip.

*Note: To underlay, gently insert the tapered gel loading tip on the bottom of the 4.9 mL OptiSeal tube and slowly pipette out each layer, careful to avoid pipetting any bubbles.*

20. Underlay again with 1.5 mL of the 20% iodixanol solution (from step D16) containing the homogenate.

21. Top off with 500 μL of HBS (see Recipe 15) so that no air remains in the tube and seal with the provided cap.

22. Load tubes in a VTi65.2 rotor and centrifuge at 60,000 rpm (327,000× *g*) for 3 h in a Beckman Optima XL 80K ultracentrifuge set to 4 °C with maximum acceleration and deceleration rates.

23. Following centrifugation, carefully pierce a hole in the bottom of the OptiSeal tube with a thumbtack and collect the desired number of fractions in a clean microcentrifuge tube. Store on ice.

24. Using a Bausch & Lomb refractometer, measure the refractive index of each fraction by adding 30 μL of each fraction to the prism while the light is on.

25. Using the focus knobs, align the semi-circle of light such that it bisects the middle of the crosshair markings visible in the eyepiece.

26. Turn on the switch on the back of the refractometer to illuminate the scale bars and record the refractive index of the fraction. Clean the prism with a Kimwipe and repeat.

27. Calculate solution density using the following formula:

Density = 3.3508 × (refractive index) - 3.4675

28. While the fractions are fresh, it is best to perform the LipoGlo electrophoresis assay (section C) as soon as possible.

**E. LipoGlo microscopy imaging**

1. Fix larvae in room-temperature 4% PFA diluted in PBS (see Recipe 19) for 3 h at room temperature.

2. Rinse larvae three times in PBS containing 0.1% Tween-20 for 15 min each.

3. Larvae should be imaged within the next 12 h to capture the maximum luminescence.

4. Prepare low-melt agarose with 0.1 g of low-melt agarose in 10 mL of 1× TBE and make 1 mL aliquots or smaller.

5. Keep low-melt agarose aliquots in liquid form at 42 °C in a heat block.

6. Just prior to mounting, supplement the low-melt agarose aliquot with 1% LipoGlo substrate (add 1 μL of substrate per 100 μL of low-melt agarose aliquot).

7. On a Petri dish lid, pipette an individual fixed larvae from the wash solution into a droplet and remove excess liquid with a pipette.

8. Quickly cover the larvae with a 50 μL droplet of low-melt agarose supplemented with 1% LipoGlo substrate.

9. Orient larvae as desired with a flexible poker until agarose is solidified. For most purposes, larvae will be arrayed laterally with the head pointed to the left.

10. Repeat this process for up to five larvae on one Petri dish lid before imaging.

11. Image using a Zeiss AxioZoom V16 with ZEN imaging software (version 2.5), equipped with a Zeiss AxioCam MRm set to the following specifications:

a. 30× magnification.

b. 2 × 2 binning.

c. 2× gain (to increase sensitivity).

d. Program to collect a single brightfield exposure (2.4 ms, 10% light intensity).

e. Follow with two chemiluminescent images (10 and 30 s, respectively).

12. Save images for subsequent analysis using Fiji.

**Caution:** For the best images, eliminate as many sources of light as possible in the imaging room being used. For example, any sources of light on the microscope should be covered with aluminum foil, and the entire microscope should be wrapped in a black curtain; room lights and computer screens should be turned off for all imaging.

**Caution:** To overcome the high background with the long exposure, place a Zeiss BG40 IR blocking filter in front of the camera. This step is especially important as there is an LED inside the light path in this microscope.

## Data analysis

**A. LipoGlo counting assay analysis**

To measure the difference in LipoGlo quantities from the counting assay, import the data obtained from the plate reader into GraphPad Prism or other graphing software, and use the group or column plot tool to visualize them graphically. See Figure 1 for an example.

*Note: For larval samples older than 7 dpf, or if fed exogenously, normalize the data using a Bradford protein assay.*

**B. LipoGlo electrophoresis assay analysis**

To quantify the different sizes of lipoproteins on the PAGE gels, we applied the following step-by-step procedure:

1. Open **File S1**: PAGE_gel_analysis_templet.xlsx.

2. After collecting the PAGE gel images, open the 600.tif and Chemi.tif images on a computer or laptop with Fiji installed by clicking *Plugins* > *Bio-formats* > *Bio-formats importer* > navigate to the 600.tif file and select *open*.

3. Repeat for the Chemi.tif file.

4. Adjust the display on the 600.tif file so the outline of the bottom glass plate is visible by clicking *Image* > *Adjust* > *Brightness/contrast* and slide the maximum bar to the left until the bottom glass plate is visible.

5. Rotate the 600.tif image so the short side of the glass plate is perfectly vertical by clicking *Image* > *Transform* > *Rotate* > Select *preview* to get crosshairs on the image. Once the image is square, record the *Angle* value in the *Gel image rotation (degree)* box in the PAGE_gel_analysis_templet.

6. Enable the *Vertical Profile* option by clicking *Edit* > *Options* > *Plots* and select *Vertical Profile*.

7. On the 600.tif image, draw a region of interest (ROI) around the DiI-LDL sample column.

8. Select *Edit* > *Selection* > *Specify* and set the height to 500; if a 10-well comb was used, set the width to 50; if a 15-well comb was used, set the width to 30.

9. Using the arrow keys, move the ROI so the top of the box is in the middle of the loading well for the DiI-LDL, and arrow up 20 key strokes.

10. Select *Edit > Selection > Specify* and record the Y-axis value from this box in the Excel worksheet in the “ROI Y-coordinate (pixels)” box.

11. Select *Analyze > Plot profile > List > Edit > Select all > Edit* and copy and paste these values into the first two rows under the “Paste ladder + raw data” table.

12. Now, working with the Chemi.tif file, adjust the brightness of the image to see all the lanes; then, rotate the image using the recorded ‘Angle’ value from the 600.tif image in the “Gel image rotation (degree)” box in the PAGE_gel_analysis_templet.

13. On the Chemi.tif image, draw a region of interest (ROI) around the first sample column.

14. Select *Edit > Selection > Specify* and set the height to 500; if a 10-well comb was used, set the width to 50; if a 15-well comb was used, set the width to 30.

15. Set the y-coordinate to the value recorded for the 600.tif image.

16. Using the arrow keys, move the ROI so the top of the box is in the middle of the loading well for the first sample, and arrow up 20 key strokes.

*Note: The y-coordinate for the ROI does not change.*

17. Select *Analyze > Plot profile > List > Edit > Select all > Edit* and copy and paste these values into the next rows under the “Paste ladder + raw data” tab in the Excel worksheet.

18. Use the arrow keys to move the ROI box rightward and center it on the second sample lane. Select *Analyze > Plot profile > List > Edit > Select all > Edit* and copy and paste these values into the next rows under the “Paste ladder + raw data” tab in the Excel worksheet. Repeat this process for all sample lanes.

19. When all plot profiles are pasted into the “Paste ladder + raw data” tab, select all the “Distance_microns” columns and delete them.

20. Select all remaining “Gray_value” columns and copy and paste them into the “Calculations” tabs, starting in A1.

21. This will allow for the automatic population of the remaining boxes in the “Calculations” table.

22. These data can then be graphed in Prism using a group chart type.

*Note: The ZM was left in* ***File S1***: *PAGE_gel_analysis_templet.xlsx, but it is suggested not to include it when graphing the results.*

**C. LipoGlo microscopy analysis**

**Figure 2.**
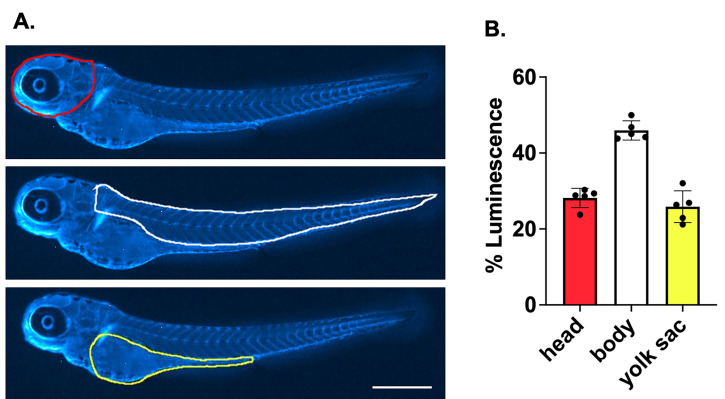
LipoGlo microscopy assay: luminescence quantified from different anatomical regions of a 3-day post-fertilization (dpf) zebrafish larvae. (A) Fiji can be used to outline regions of interest (ROI) and measure the total luminescence in the defined region. The red ROI is the head, white is the body, and yellow is the yolk sac region. Scale bar, 500 μm. (B) Total percent luminescence for each ROI (N = 2, n = 5 larvae analyzed for each ROI). Error bars indicate overall mean ± SD of two independent experiments.

1. Open the whole-mount images with Fiji.

2. Open the ROI manager by selecting Analyze > Tools > ROI Manager.

3. On the image, use the freehand selection tool to trace the ROI (Figure 2A) and add it to the ROI manager by selecting Add on the ROI Manager window.

4. Once all ROIs have been defined, select all of them and click “Measure” to get a popup window with the following measurements:

a. Area

b. Mean

c. IntDen

d. RawIntDen

5. Copy and paste all values into a new Excel sheet for calculating the total percent luminescence for each ROI.

6. For each set of ROIs, calculate the sum total of the RawIntDen values and divide each individual ROI’s RawIntDen value by the sum total to get the total percent luminescence for each ROI.

7. These values can then be graphed using Prism or other graphing software (Figure 2B).

**Critical:** The total in this analysis will not sum to 100% because the heart was not included in any of the ROIs for this experiment.

## Validation of protocol

To interrogate and validate our updated protocol, we began by optimizing the amount of LipoGlo substrate needed in a typical LipoGlo counting assay. These data are required to determine if sufficient substrate is available for typical assay amounts and the time required to perform the plate reader assay. For these experiments, 4-dpf larvae homozygous for the LipoGlo reporter were used. Each LipoGlo plate was read every 15 min for the duration of this experiment (1 h and 5 min) using varying amounts of homogenate (40, 20, 10, and 4 μL). From these data, 4 μL of homogenate provided the best LipoGlo counting assay performance (Figure 1A), producing the most linear response over the time points measured (Figure 1B). Next, we aimed to verify that 4 μL of homogenate, compared with the original 40 μL, was ideal when measuring total ApoB-LPs over zebrafish larval development from 1 to 7 dpf. Homogenate from 4-dpf larvae homozygous for the reporter was used at 40 or 4 μL in a LipoGlo counting assay for each day of larval development from 1 to 7 dpf (Figure 1C). These data indicate that using 4 μL of homogenate is superior to using 40 μL of homogenate for the LipoGlo counting assay.

**Figure 3.**
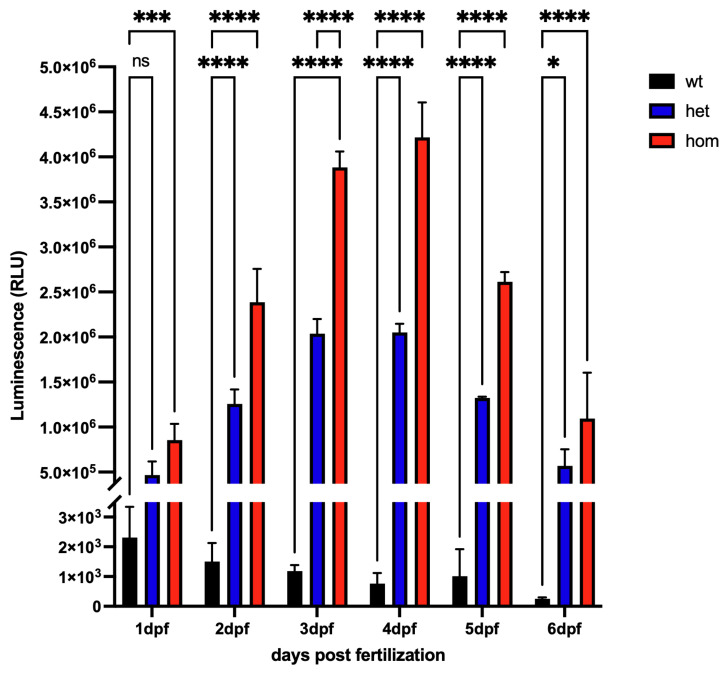
LipoGlo counting assay: zebrafish larvae homozygous for the LipoGlo reporter have a 2-fold increase in Apolipoprotein B–containing lipoproteins (ApoB-LPs). Zebrafish heterozygous for the ApoBb.1 reporter are in blue (het), homozygous animals are in red (hom), and wild type are in black (wt). There is nearly a 2-fold increase in luminescence between heterozygous and homozygous animals at each day of development measured. N = 3, n = 8–52. Error bars indicate overall mean ± SD of three independent experiments. Two-way ANOVA with Šidák’s multiple comparison test, *p < 0.0269, ***p < 0.0002, ****p < 0.0001, ns, non-significant.

Next, we wanted to measure the levels of ApoB-LPs in the three genotypes of our reporter line, *Fus(ApoBb.1-nluc)*. For each day of development (1–6 dpf), a 96-well plate of larval homogenate from an in-cross of *Fus(ApoBb.1-nluc)^+/-^* adults was used for a LipoGlo counting assay. To confirm the linearity of the assay response and ensure that sufficient substrate is available to accommodate a 2-fold increase in enzyme levels, we compared 4 μL of homozygous *Fus(ApoBb.1-nluc)-/-* larvae homogenate to 4 μL of heterozygous sibling homogenate. Each 96-well plate was genotyped for the presence of *ApoBb.1-nluc* (e.g., WT, heterozygous *ApoBb.1-nluc* and homozygous *ApoBb.1-nluc*). A 2-fold increase of ApoB-LP luminescence was seen with each day of development (Figure 3), confirming that the assay is linear for at least a 2-fold increase in enzyme.

**Figure 4.**
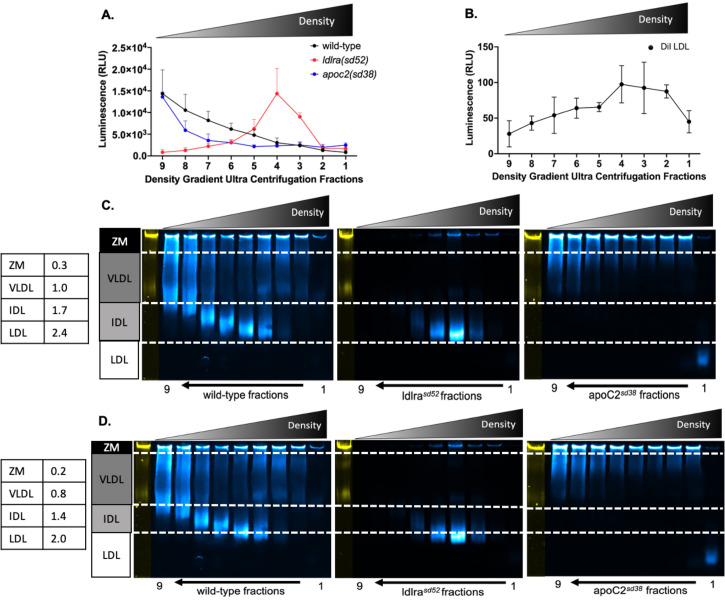
Density gradient ultra-centrifugation (DGUC) assay: validating the use of human DiI-LDL as the standard control and PAGE gel analysis recalibration. (A) LipoGlo apolipoprotein B–containing lipoprotein (ApoB-LP) count assays from DGUC fractions 9–1 for wild-type 4 dpf, *ldlra^sd52^* 7dpf and *apoC2^sd36^* 7 dpf mutant zebrafish lines, N = 3 clutches, n = 15 larvae/clutch. Error bars indicate overall mean ± SD of two independent experiments (B) Fluorescent plate read assay with DGUC fractions collected from the human DiI-LDL migration standard, N = 2. (C) LipoGlo electrophoresis results showing the nine fractions for 5-dpf wild-type larvae, 7-dpf *ldlra^sd52^* larvae, and *apoC2^sd36^* 7-dpf larvae. Original cutoff bins and their associated ApoB-LP type are indicated on the left with white dashed lines. (D) LipoGlo electrophoresis results showing the nine fractions for 5-dpf wild-type larvae, 7-dpf *ldlra^sd52^* larvae, and *apoC2^sd36^* 7-dpf larvae. Recalibrated cutoff bins and their associated ApoB-LP type are indicated on the left with white dashed lines.

For the LipoGlo electrophoresis assay, it was previously noted that human DiI-LDL had a slower migration rate due to the presence of the DiI [1]. Since the development of the assay, we have observed that the human DiI-LDL band migrates differently from lot to lot. This difference in migration distance is important because it was used in the analysis to account for gel variation in the quantification of the PAGE signal used to quantify ApoB-LP particle sizes. To re-validate our assay, we first needed to verify that human DiI-LDL and zebrafish LipoGlo LDL were similar in size, and second, we needed to provide a method to recalibrate the LipoGlo electrophoresis assay when using a new lot of DiI-LDL. To address these issues, we used density gradient ultra-centrifugation (DGUC) to measure which fraction of the human DiI-LDL and zebrafish LDL was detected (if their densities/sizes are the same, they should concentrate in the same fraction). For this experiment, we used human DiI-LDL, 5-dpf *Fus(ApoBb.1-nluc)^+/+^*, 7-dpf *ldlra^sd52^* zebrafish mutants enriched in small LDL particles, and 7-dpf *apoC2^sd38^* zebrafish mutants with high levels of large VLDL particles. The time points selected for sampling were determined as when each fish had the strongest ApoBb.1 phenotype. The DGUC assay was performed as described in section D, and 10 fractions were collected for each sample. When the fractions were subjected to the LipoGlo electrophoresis assay, one key observation was that the LDL fractions for *ldlra^sd52^* and the human DiI-LDL both eluted in the fourth fraction (Figure 4A, 4B). To recalibrate the LipoGlo size analysis, we ran all the fractions on a PAGE gel and observed that when the original cutoff bins from the publication were used, there was obvious underrepresentation of LDL (Figure 4C). When the cutoff bins were manually adjusted to reflect where the *ldlra^sd52^* mutants’ small particles ran and where the *apoC2^sd38^* mutants’ large particles ran (Figure 4D), all ApoB-LP classes were more accurately measured.

In the original publication describing the LipoGlo electrophoresis assay [1], we always observed LipoGlo signal at the base of the loading well, which we referred to as the zero-mobility (ZM) fraction. We assumed that this fraction was composed of a mixture of tissue aggregates and extremely large lipoproteins like those produced by the intestine after a high-fat meal (chylomicrons). We now know that following DGUC of tissue extracts, each size fraction produces a zero-mobility signal. These data are consistent with aggregation occurring when the gel is run, since it is occurring in each fraction (Figure 4D), representing 20%–40% of the signal in each fraction. Fractions with larger particles have a higher percentage of zero mobility signal than those from the smallest particle fractions (see **File S2**). While the ZM fraction is calculated in our **File S1**: PAGE_gel_analysis_templet.xlsx, it might be best to exclude it when describing and graphing the distribution of particle sizes, since it likely represents aggregates from all size fractions.

## General notes and troubleshooting

**Table:** 

Common problems encountered	Solutions
Not all larvae are completely sonicated during the sonication procedure.	When possible, avoid using the outermost wells, as these are the wells most likely not to fully sonicate. Try turning the plate to a 45° angle (i.e., changing the location of the unsonicated wells within the sonicator horn). Using additional sonication pulses can sometimes shear the DNA into very small fragments, which can make downstream PCR challenging.
No amplicons in PCR using gDNA made from the homogenate.	When designing primers for PCR, target a 200 bp or smaller PCR product.
PAGE gels are sticking to the back plate for imaging and/or have wavy lane borders.	Treat both sides of the front plate with Rain-X; this allows for the front plate to be removed without distorting the PAGE gel. Treat front plates every 3–4 uses.
LipoGlo microscopy images exhibit high levels of nonspecific background signal.	Be sure to eliminate all sources of light in the room used for imaging. Specifically, turn off overhead lights, turn off the computer screen during imaging, use aluminum foil to cover light sources (e.g., LED indicator lights), and drape the microscope in a black cloth. Be sure that all fluorescent light sources associated with the imaging room are powered off. We have found that the Zeiss AxioZoom V16 contains infrared emitters and detectors within the imaging path, which result in a very high background when long exposures are used. To overcome this issue, we placed a Zeiss BG40 IR blocking filter in front of the camera, which effectively filtered the contaminating infrared light.
LipoGlo microscopy images are faint.	The absolute intensity of the NanoLuc signal will decay gradually over time. To achieve the strongest signal, it is recommended that larvae be mounted and imaged with an incubation period as short and consistent as possible (ideally <30 min).

## Supplementary information

The following supporting information can be downloaded here:

1. LipoGlo PAGE Analysis Template (File S1: PAGE_gel_analysis_templet.xlsx)

2. Density gradient PAGE gel analysis (File S2: PAGE_gel_DGUC_Analysis.xlsx)
